# Polarization of the Majorana quasiparticles in the Rashba chain

**DOI:** 10.1038/s41598-017-16323-3

**Published:** 2017-11-23

**Authors:** Maciej M. Maśka, Tadeusz Domański

**Affiliations:** 1 Institute of Physics, University of Silesia, 40-007 Katowice, Poland; 2 Institute of Physics, M. Curie-Skłodowska University, 20–031 Lublin, Poland

## Abstract

We demonstrate that the selective equal–spin Andreev reflection (SESAR) spectroscopy can be used in STM experiments to distinguish the zero–energy Majorana quasiparticles from the ordinary fermionic states of the Rashba chain. Such technique, designed for probing the *p*–wave superconductivity, could be applied to the intersite pairing of equal–spin electrons in the chain of magnetic Fe atoms deposited on the superconducting Pb substrate. Our calculations of the effective pairing amplitude for individual spin components imply the magnetically polarized Andreev conductance, which can be used to ‘filter’ the Majorana quasiparticles from the ordinary in–gap states, although the pure spin current (i.e., perfect polarization) is impossible.

## Introduction

The topologically nontrivial superconducting state of one–dimensional (1D) chains^1^ allows for a unique phenomenon of the *selective equal–spin Andreev reflection* (SESAR). This polarized Andreev spectroscopy has been proposed by J. J. He *et al*.^2^ as a useful tool for probing the Majorana states. SESAR measurements have indeed provided evidence for the zero–energy modes in vortices of the *p*–wave superconducting Bi_2_Te_3_/NbSe_2_ heterostructures^3,4^. Similar ideas have been also considered for the Josephson–type junctions^5,6^ and ferromagnet–superconductor interfaces with the spin–orbit coupling^7,8^. In this work we demonstrate that SESAR spectroscopy can test *inherent polarization* of the Majorana quasiparticles appearing at the edges of the Rashba chain. The parallel and perpendicular components of magnetically polarized Majorana states has initially been pointed out by D. Sticlet *et al*.^9^ and their signatures have been recently studied by a number of authors^10–13^. In this paper we show that magnetic polarization is detectable in STM experiments owing to SESAR processes, which in the subgap regime could distinguish the Majorana quasiparticles out the ordinary Shiba in-gap states. We provide microscopic arguments explaining such polarization and confront our predictions with the experimental data obtained for Fe atom chain deposited on the surface of Pb superconductor by the STM technique with use of the magnetically polarized tip^14^.

**Figure 1:**
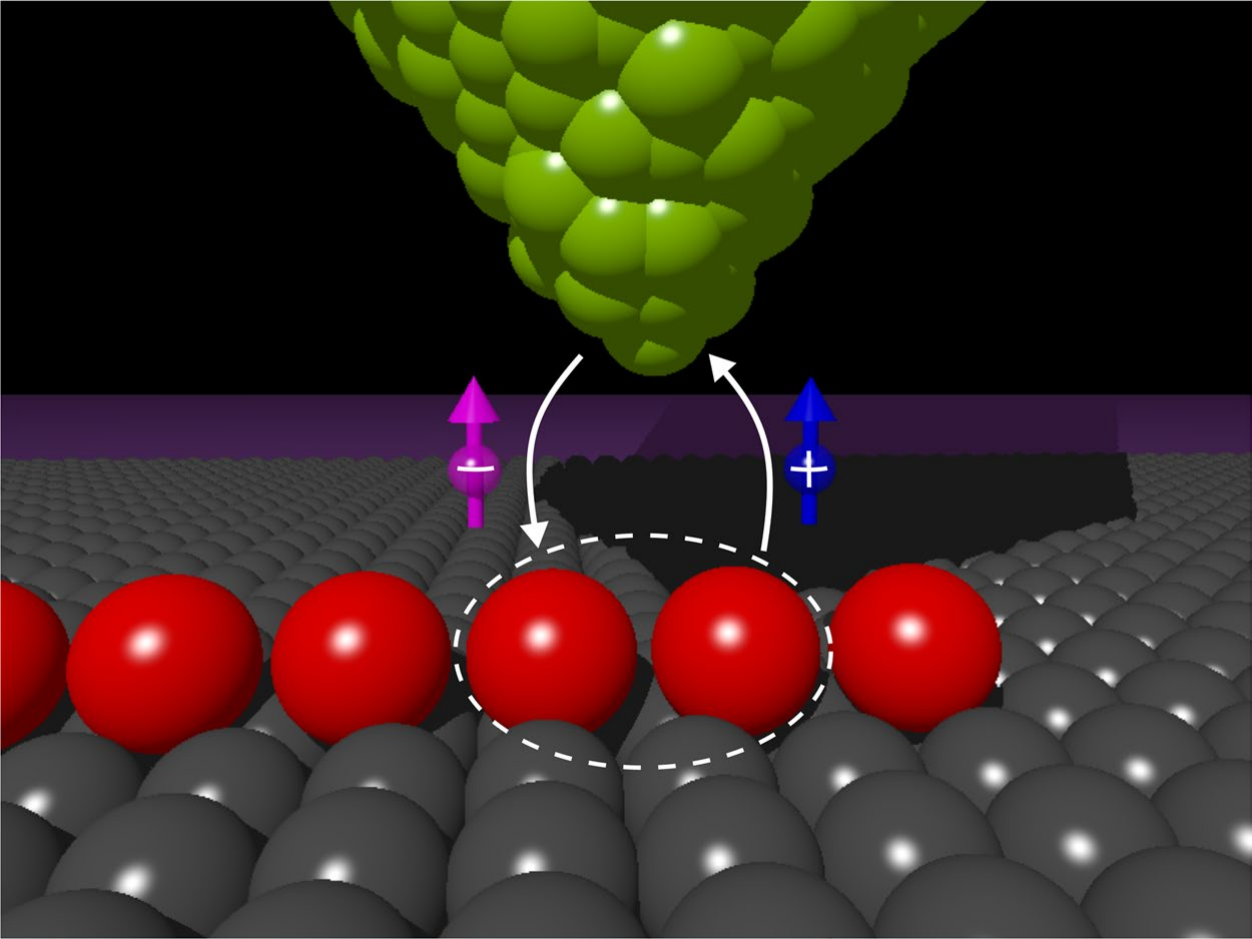
Schematic idea of SESAR. This polarized Andreev spectroscopy can probe the intersite pairing (represented by the dashed ellipse) of electrons on Fe atoms (red color) deposited on the *s*–wave bulk superconductor (gray) by using the magnetically polarized STM tip (green color).

The underlying idea of SESAR for the aforementioned configuration is displayed in Fig. 1. This STM–type setup has been previously used by several experimental groups^15–17^, however, ignoring the magnetic polarization. Recently A. Yazdani and coworkers^14^ have measured the spin–resolved tunneling current and revealed substantial polarization of the zero–bias conductance in regions, where the Majorana quasiparticles exist. This fact can be interpreted within the popular microscopic model, taking into account the Rashba and Zeeman interactions in addition to the proximity–induced pairing which can realistically capture a topography of the Majorana fermions^9,18–20^. Using this model we have recently emphasized^21^, that amplitude of the intersite pairing (between identical spin electrons) differs several times for ↑ and ↓ sectors, respectively. Obviously, such effect should give rise to noticeable polarization of the Majorana quasiparticles near the chain edges. In practice, the low–energy features can be detected only by the anomalous Andreev spectroscopy, as discussed in detail in ref.^18^. Since efficiency of the particle to hole conversion for the spin–polarized Andreev spectroscopy depends on the anomalous propagator ${\langle \langle {d}_{i,\sigma };{d}_{i+\mathrm{1,}\sigma }\rangle \rangle }_{\omega +i{0}^{+}}$, one should expect its non–vanishing value at *ω* = 0 nearby the chain edges. In what follows we show, that this is really the case. We also argue, that SESAR could distinguish the Majorana from the ordinary fermionic quasiparticles.

## Results

### Microscopic model

Nanoscopic chain of the magnetic Fe atoms deposited on the *s*–wave conventional superconductor and probed by the polarized STM tip (relevant to the experimental situation^14^) can be described by the Hamiltonian^9,18–20^ $\hat{H}={\hat{H}}_{{\rm{tip}}}+{\hat{V}}_{{\rm{tip}}-{\rm{chain}}}+{\hat{H}}_{{\rm{chain}}}+{\hat{V}}_{{\rm{chain}}-{\rm{S}}}+{\hat{H}}_{{\rm{S}}}$. We treat the STM tip ${\hat{H}}_{{\rm{tip}}}$ as a free fermion gas and focus on quasiparticle states of the atomic chain appearing deep inside the superconducting gap. Under such circumstances the superconducting reservoir would be responsible for the proximity induced on-site pairing ${\hat{H}}_{{\rm{chain}}}+{\hat{V}}_{{\rm{chain}}-{\rm{S}}}+{\hat{H}}_{{\rm{S}}}\to {\hat{H}}_{{\rm{chain}}}^{{\rm{prox}}}$ (for technical details see, e.g., Appendix A in ref.^21^). In what follwos, we impose the constant couplings Γ_*N*_ and Γ_*S*_ to the STM tip and superconducting substrate, respectively (see Fig. 1).

The low–energy Hamiltonian is effectively given by^19^

1 $${\hat{H}}_{{\rm{chain}}}^{{\rm{prox}}}=\sum _{i,j,\sigma }\,({t}_{ij}-\mu {\delta }_{i,j}){\hat{d}}_{i,\sigma }^{\dagger }{\hat{d}}_{j,\sigma }+{\hat{H}}_{{\rm{prox}}}+{\hat{H}}_{{\rm{Rashba}}}+{\hat{H}}_{{\rm{Zeeman}}},$$

where ${\hat{d}}_{i,\sigma }^{(\dagger )}$ annihilates (creates) an electron with spin *σ* at site *i*, *t*_*ij*_ is the hopping integral and *μ* is the chemical potential. The proximity effect, responsible for the on–site (trivial) pairing, can be modeled as^20^

2 $${\hat{H}}_{{\rm{prox}}}={\rm{\Delta }}({\hat{d}}_{i,\uparrow }^{\dagger }{\hat{d}}_{i,\downarrow }^{\dagger }+{\hat{d}}_{i,\downarrow }{\hat{d}}_{i,\uparrow })$$

with the pairing potential Δ = Γ_*S*_/2. In this scenario the intersite *p*–wave pairing is driven by the Rashba and the Zeeman interactions

3 $${\hat{H}}_{{\rm{Rashba}}}=-\alpha \,\sum _{i,\sigma ,\sigma ^{\prime} }\,[{\hat{d}}_{i+\mathrm{1,}\sigma }^{\dagger }{(i{\sigma }^{y})}_{\sigma \sigma ^{\prime} }{\hat{d}}_{i,\sigma ^{\prime} }+{\rm{H}}{\rm{.c}}{\rm{.}}],$$

4 $${\hat{H}}_{{\rm{Zeeman}}}=\frac{g{\mu }_{{\rm{B}}}B}{2}\,\sum _{i,\sigma ,\sigma ^{\prime} }\,{\hat{d}}_{i,\sigma }^{\dagger }{({\sigma }^{z})}_{\sigma {\sigma }^{^{\prime} }}{\hat{d}}_{i,{\sigma }^{^{\prime} }}\mathrm{.}$$

We assume the magnetic field to be aligned along $\hat{z}$–axis and impose the spin–orbit vector ***α*** = (0, 0, *α*).

### Spin–polarized Majorana quasiparticles

**Figure 2:**
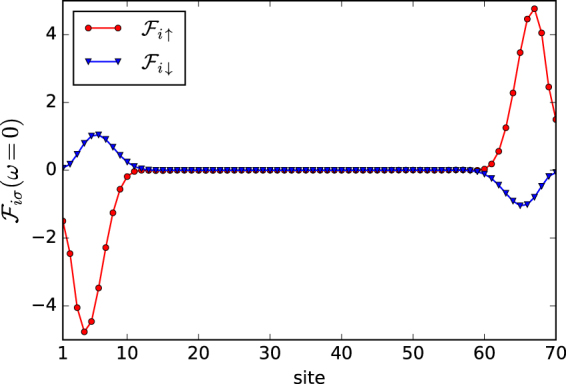
Intrinsic polarization of Majorana quasiparticles. The off–diagonal spectral function *F*_*iσ*_(*ω*) obtained at zero energy (*ω* = 0) for the inter–site pairing of *σ* spin electrons, using Δ = 0.2 *t*, *α* = 0.15 *t*, *μ* = −2.1 *t*, and *gμ*_*B*_ *B*/2 = 0.27 *t*.

In Fig. 2 we present spatial dependence of the off–diagonal spectral function ${ {\mathcal F} }_{i\sigma }(\omega )=-\frac{1}{\pi }\,{\rm{Im}}\,{\langle \langle {\hat{d}}_{i,\sigma };{\hat{d}}_{i+\mathrm{1,}\sigma }\rangle \rangle }_{\omega +i{0}^{+}}$ obtained at zero energy for different spins ↑ and ↓, respectively. This anomalous spectral function is very instructive, because its sign exhibits *intrinsic polarization* of the Majorana modes (previously emphasized in ref.^9^) whereas its absolute value can be probed by the SESAR spectroscopy (see the next paragraph). Concerning the magnitude, we clearly notice a quantitative difference (almost 5 times) between the spin ↑ and ↓ inter–site pairings. As regards the intrinsic polarization we observe that *F*_*iσ*_(*ω* = 0) changes its phase by *π* between opposite sides of the Rashba chain and furthermore each of the spin sectors is characterized by opposite polarizations. This aspect resembles the results reported for the interface of ferromagnet/superconductor bilayers^22^. Such feature can be regarded as a hallmark of the finite–size systems, because otherwise (i.e., in thermodynamic limit *L* → ∞) the off–diagonal spectral function would identically vanish at zero energy for both pairing channels.

**Figure 3:**
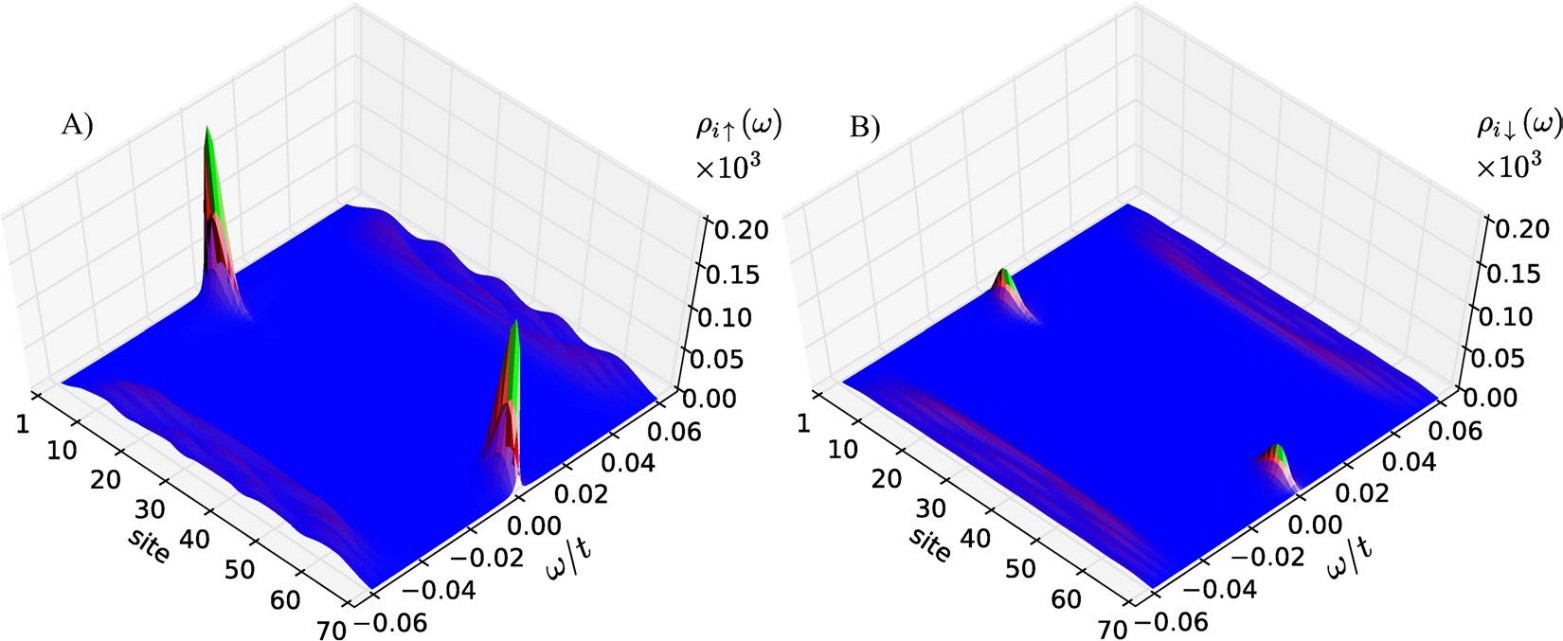
Topography of the polarized quasiparticles. The spin–up (**A**) and spin–down (**B**) (diagonal) spectral functions *ρ*_*iσ*_(*ω*) determined at low energies which reveal, that the zero–energy (Majorana) quasiparticles are strongly polarized.

Figure 3 illustrates the spatial profiles of the spin–polarized (diagonal) spectral function *ρ*_*iσ*_(*ω*). As expected, we notice quantitative differences between the Majorana states appearing in ↑ and ↓ spin sectors, whereas their overall profiles seem to be pretty similar. Different magnitudes of these spin–polarized Majorana quasiparticles would show up in the SESAR measurements.

### The polarized Andreev transport

By applying a bias voltage *V* between the STM tip and the superconducting reservoir one would induce the nonequilibrium charge transport. Deep in a subgap regime (i.e., for $|V|\ll {\rm{\Delta }}/|e|$) such current is contributed solely by the Andreev scattering, when electrons from the STM tip are converted into the pairs, reflecting holes back to the STM tip. This process can be treated within the Landauer–Büttiker formalism.

We can express the nonmagnetic (*γ* = 0) and magnetically polarized (*γ* = *σ*) Andreev currents by the following formula

5 $${I}_{i}^{\gamma }(V)=\frac{e}{h}\,\int \,d\omega \,{T}_{i}^{\gamma }(\omega )\,[f(\omega -eV)-f(\omega +eV)],$$

where *f*(*x*) = [1 + exp(*x*/*k*_*B*_ *T*)] stands for the Fermi–Dirac distribution function. These Andreev channels are characterized by various (dimensionless) transmittances, that can be expressed via the local and non–local anomalous Green’s functions, respectively

6 $${T}_{i}^{0}(\omega )={{\rm{\Gamma }}}_{N}^{2}\,({|\langle \langle {\hat{d}}_{i\uparrow };{\hat{d}}_{i\downarrow }\rangle \rangle |}^{2}+{|\langle \langle {\hat{d}}_{i\downarrow };{\hat{d}}_{i\uparrow }\rangle \rangle |}^{2}),$$

7 $${T}_{i}^{\sigma }(\omega )={{\rm{\Gamma }}}_{N}^{2}\,({|\langle \langle {\hat{d}}_{i\sigma };{\hat{d}}_{i+1\sigma }\rangle \rangle |}^{2}+{|\langle \langle {\hat{d}}_{i\sigma };{\hat{d}}_{i-1\sigma }\rangle \rangle |}^{2})\mathrm{.}$$

Exceptionally, for the edge sites *i* = 1 and *i* = *L* the spin polarized transmittance is ${T}_{1}^{\sigma }(\omega )={{\rm{\Gamma }}}_{N}^{2}\,{|\langle \langle {\hat{d}}_{1\sigma };{\hat{d}}_{2\sigma }\rangle \rangle |}^{2}$ and ${T}_{L}^{\sigma }(\omega )={{\rm{\Gamma }}}_{N}^{2}\,{|\langle \langle {\hat{d}}_{L\sigma };{\hat{d}}_{L-1\sigma }\rangle \rangle |}^{2}$. Derivation of formula (6) is presented in section Methods. These off-diagonal Green’s functions can be computed numerically from the Bogoliubov–de Gennes treatment of the Rashba chain (1). Obviously, in experiments with the unpolarized STM tip^15,16^ the total current contains all three components, i.e. ${I}_{i}(V)={\sum }_{\gamma }\,{I}_{i}^{\gamma }(V)$.

**Figure 4:**
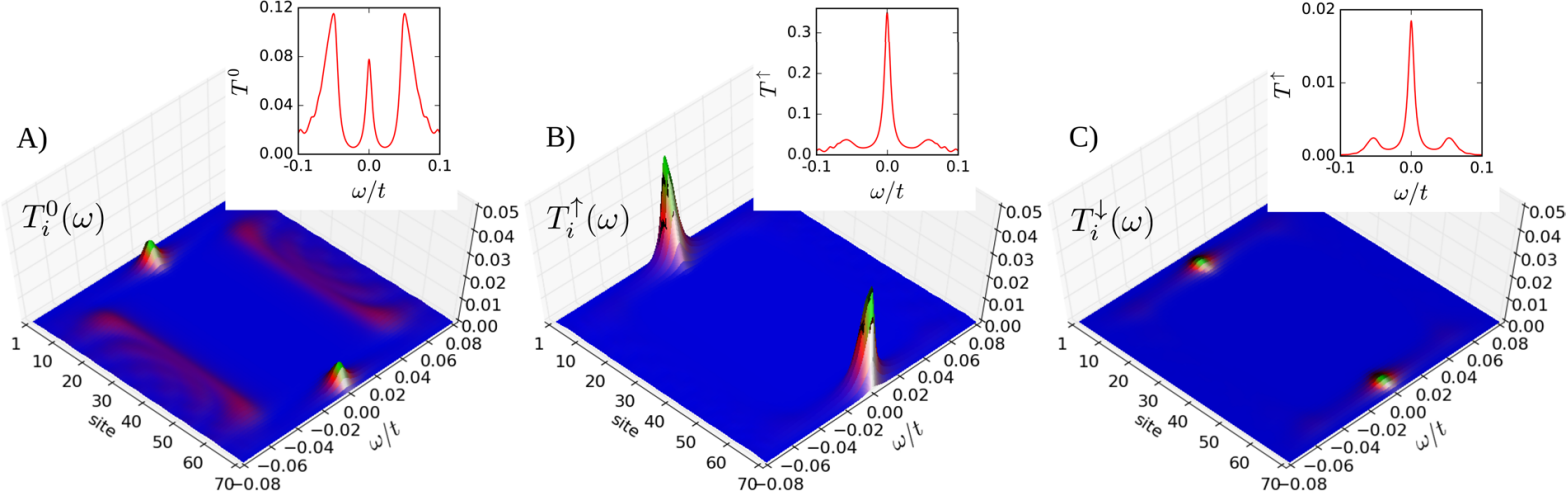
Subgap transmittances. The spatially resolved transmittances ${T}_{i}^{\gamma }(\omega )$ obtained at low energies $(|\omega |\ll {\rm{\Delta }})$ for the nonmagnetic *γ* = 0 (panel (A)) and the spin–polarized Andreev reflections *γ* = ↑ (panel (B)) and *γ* = ↓ (panel (C)). The insets display the transmittances summed over all lattice sites.

Figure 4 shows the energy–dependent transmittances ${T}_{i}^{\gamma }(\omega )$ obtained for the non–polarized (*γ* = 0) and spin–polarized (*γ* = *σ*) Andreev channels. The difference between unpolarized and polarized transmittances is especially visible in the insets, where ${T}^{\gamma }(\omega )\equiv {\sum }_{i}\,{T}_{i}^{\gamma }(\omega )$ is plotted. In the case of *T* ^0^(*ω*) the ordinary (finite-energy) Shiba states are are showing up (panel A), whereas in the polarized transmittances *T* ^↑,↓^(*ω*) the Majorana quasiparticle plays the clearly dominat role (panels B and C).

**Figure 5:**
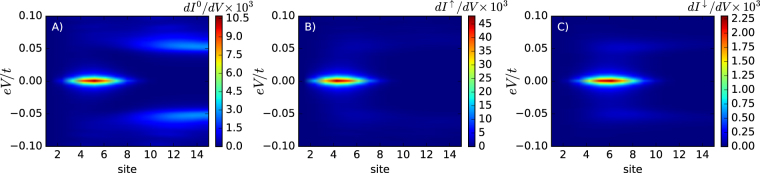
Subgap conductances. False color plots of the differential conductance $d{I}_{i}^{\gamma }(V)/dV$ of the ordinary (*γ* = 0, panel (A)) and the spin–resolved (*γ* = ↑, panel (B) and *γ* = ↓, panel (C)) Andreev transport channels obtained at temperature *T* = 5 · 10^−4^ *t*. The conductance is expressed in units 4*e* ^2^/*h*. Plots (B) and (C) look very similar, but notice a strong difference in their scales.

The corresponding conductances are presented in Fig. 5. We notice that the differential conductance of the nonmagnetic Andreev reflections dominates well inside the Rashba chain at energies coinciding with the fermion Andreev/Shiba states. The SESAR, on the other hand, is efficient mainly near the Majorana modes whose spatial extent covers roughly 10 sites near the Rashba chain edges. In distinction to ref.^2^, we observe that the spin–polarized currents are present for both spins (↑ and ↓) but with significantly different magnitudes. Our results are relevant to the recent experimental data reported by the Princeton group^14^. We have checked that the spin–polarized Majorana quasiparticles are robust upon varying the model parameters, although some additional subtle effects may be observed, for instance the quantum oscillations^18^.

**Figure 6:**
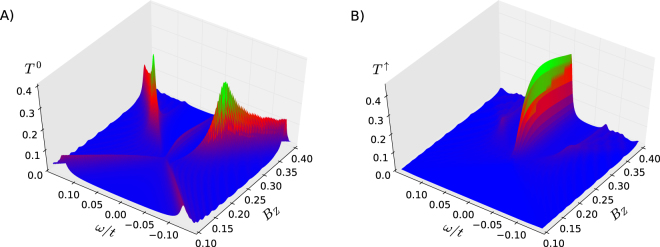
Evolution of transmittances. Unpolarized *T* ^0^(*ω*) (Panel (A)) and polarized *T* ^↑^ (*ω*) (Panel (B)) transmittances summed over all lattice sites as a function of magnetic field. The topological phase starts around *B*_*Z*_ = 0.21.

The results presented in Fig. 5 correspond to the topological regime. By varying the model parameters so that the system is driven to the topologically trivial phase, the zero-energy Majorana peak vanishes and the total transmittance in the spin-polarized channels is strongly suppressed. Such evolution from the topologically trivial to nontrivial state is presented in Fig. 6. Note that the polarized transmittance *T* ^↑^ (*ω*) vanishes almost completely outside the topological regime. In the topological regime the unpolarized transmittance of the Majorana peak is much smaller than the transmittance of the ordinary in-gap states that develop when the system enters the topological regime. On the other hand, the polarized transmittance of the Majorana peak is much larger than the ordinary in-gap states.

In summary, we emphasize that the net spin current ${I}_{i}^{{\rm{spin}}}(V)={I}_{i}^{\uparrow }(V)-{I}_{i}^{\downarrow }(V)$, attainable from the SESAR spectroscopy, is expected to acquire meaningful values of the spatially–resolved conductance ${G}_{i}^{{\rm{spin}}}(V)=\partial {I}_{i}^{{\rm{spin}}}(V)/\partial V$ only near the Majorana quasiparticles (what can be inferred by inspecting Fig. 5). SESAR can hence *filter* the Majorana from the ordinary Andreev/Shiba quasiparticles (which always exist in the Rashba chain). This unique virtue of SESAR would be valuable for spotting the Majorana quasiparticles and investigating their topography.

## Discussion

We have studied the selective equal–spin Andreev spectroscopy (SESAR) which can empirically detect the polarized Majorana quasiparticles appearing at the edges of the Rashba chain. We have shown that different amplitudes of the inter–site equal–spin pairing imply the magnetic polarization of the Majorana states and yields the spin–dependent Andreev transport with substantially distinct probabilities in each spin components. Our theoretical results qualitatively agree with the recent finding by A. Yazdani^14^, who reported the spin–polarized features in the subgap spectroscopy. Even though the pure spin current (discussed in ref.^2^) is impossible – the spin current conductance ${G}_{i}^{{\rm{spin}}}(V)$ could nevertheless *filter* the Majorana quasiparticles from the ordinary Andreev/Shiba states. Our quantitative estimations clearly show also that the non–polarized and spin–polarized Andreev conductances are much smaller than the unitary limit value 2*e* ^2^/*h* as has been indeed observed by the STM^15,16,23^ and by the tunneling measurements via heterojunctions^24^.

## Methods

Our calculations have been performed for the Rashba chain, comprising *L* = 70 atoms. In most of the numerical calculations (except Fig. 6) we have used the following model parameters: magnitude of the induced pairing Δ = 0.2 *t*, the spin–orbit coupling *α* = 0.15 *t*, the chemical potential *μ* = −2.1 *t*, and the external magnetic field *gμ*_*B*_ *B*/2 = 0.27 *t*. Such a choice of parameters locates the system strictly in a topological regime^21^. The spin–resolved spectral functions, presented in Fig. 3, have been calculated using the following definition

8 $${\rho }_{i\sigma }(\omega )=-\frac{1}{\pi }\,{\rm{Im}}\,{\langle \langle {\hat{d}}_{i,\sigma };{\hat{d}}_{i,\sigma }^{\dagger }\rangle \rangle }_{\omega +i{{\rm{\Gamma }}}_{N}\mathrm{/2}},$$

where Γ_*N*_ is the coupling to the STM tip (assumed to be Γ_*N*_ = 0.01 *t*) and the Green function has been calculated numerically from $\hat{G}(\omega )={(\omega 1-{\hat{H}}_{{\rm{chain}}}^{{\rm{prox}}})}^{-1}$. For *L*–site–long chain, the Hamiltonian ${\hat{H}}_{{\rm{chain}}}^{{\rm{prox}}}$ given by Eq. (1), is 4 *L* × 4 *L* complex matrix and the currents in Eq. (5) have been calculated with a help of 8–point Gauss quadrature.

Let us outline a brief scheme for computing the charge tunneling current induced through *i*-th site of the chain coupled between the STM tip (*N* electrode) and the superconducting substrate (*S* electrode), for simplicity neglecting the inter-site hopping *t*_*ij*_ = 0. Using the Heisenberg equation we can express such current as

9 $${I}_{i}(V)=-e\frac{d}{dt}\langle {\hat{N}}_{tip}\rangle =-e\langle \frac{d}{dt}{\hat{N}}_{tip}\rangle =\frac{ie}{\hslash }\langle [{\hat{N}}_{{\rm{tip}}},{\hat{V}}_{{\rm{tip}}-i}]\rangle ,$$

where *e* stands for elementary charge, ${\hat{N}}_{{\rm{tip}}}={\sum }_{\sigma ,{\bf{k}}}\,{\hat{c}}_{{\bf{k}},\sigma }^{\dagger }{\hat{c}}_{{\bf{k}},\sigma }$ counts a number of electrons in STM tip, and ${\hat{V}}_{{\rm{tip}}-i}={\sum }_{\sigma ,{\bf{k}}}\,({V}_{{\bf{k}}}{\hat{d}}_{i,\sigma }^{\dagger }{\hat{c}}_{{\bf{k}},\sigma }+{\rm{h}}.{\rm{c}}.\,)$ denotes the hybridization of *i*-th site with itinerant electrons of the tip. Since we are interested in the spin-resolved spectroscopy let us exprees (9) as *I*_*i*_(*V*) = *I*_*i*↑_(*V*) + *I*_*i*↓_(*V*), where

10 $${I}_{i\sigma }(V)=\frac{2e}{\hslash }\,\sum _{{\bf{k}}}\,{\rm{Re}}\,\{{V}_{{\bf{k}}}{\langle \langle {\hat{d}}_{\sigma }(t);{\hat{c}}_{{\bf{k}}\sigma }^{\dagger }(t)\rangle \rangle }^{ < }\}$$

and the lesser Green’s function is defined as ${\langle \langle \hat{A};\hat{B}\rangle \rangle }^{ < }\equiv i\langle \hat{B}\hat{A}\rangle$. This mixed Green’s function can be determined using the Dyson equation ${\langle \langle \hat{A};\hat{B}\rangle \rangle }^{ < }=\langle {\{\hat{A},\hat{B}\}}_{+}\rangle +{\langle \langle [\hat{A},\hat{V}];\hat{B}\rangle \rangle }^{r}{g}^{ < }+{\langle \langle [\hat{A},\hat{V}];\hat{B}\rangle \rangle }^{ < }{g}^{a}$. In our case, we obtain

11 $$\begin{array}{rcl}{\langle \langle {\hat{d}}_{\sigma }(t);{\hat{c}}_{{\bf{k}}\sigma }^{\dagger }(t)\rangle \rangle }^{ < } & = & {V}_{{\bf{k}}}^{\ast }\,\int \,d\tau {\langle \langle {\hat{d}}_{\sigma }(t);{\hat{d}}_{\sigma }^{\dagger }(\tau )\rangle \rangle }^{r}{g}_{{\bf{k}}}^{ < }(t,\tau )\\  &  & +{V}_{{\bf{k}}}^{\ast }\,\int \,d\tau {\langle \langle {\hat{d}}_{\sigma }(t);{\hat{d}}_{\sigma }^{\dagger }(\tau )\rangle \rangle }^{ < }{g}_{{\bf{k}}}^{a}(t,\tau )\end{array}$$

with the bare Green’s functions ${g}_{{\bf{k}}}^{ < }(t,\tau )=i\,f({\varepsilon }_{{\bf{k}}}){e}^{-i({\varepsilon }_{{\bf{k}}}-eV)(t-\tau )}$ and ${g}_{{\bf{k}}}^{a}(t,\tau )=i\theta (-t+\tau ){e}^{-i({\varepsilon }_{{\bf{k}}}-eV)(t-\tau )}$.

For studying the charge transfer in the low bias regime (comparable or smaller than energy gap Δ_*sc*_ of the superconducting electrode) we can impose constant couplings to the normal ${{\rm{\Gamma }}}_{N}\equiv 2\pi \,{\sum }_{{\bf{k}}}\,{|{V}_{{\bf{k}}}|}^{2}\delta (\omega -{\varepsilon }_{{\bf{k}}})$ and superconducting electrode ${{\rm{\Gamma }}}_{S}\equiv 2\pi \,{\sum }_{{\bf{q}}}\,{|{V}_{{\bf{q}}}|}^{2}\delta (\omega -{\varepsilon }_{{\bf{q}}})$. Substituting (11) to (10) we get

12 $$\begin{array}{rcl}{I}_{i\sigma }(V) & = & -\frac{2e}{\hslash }\,\int \,\frac{d\omega }{2\pi }{{\rm{\Gamma }}}_{N}\,{\rm{Im}}\,\{{\int }_{-\infty }^{t}\,d\tau {e}^{i(\omega -eV)(t-\tau )}({\langle \langle {\hat{d}}_{\sigma }(t);{\hat{d}}_{\sigma }^{\dagger }(\tau )\rangle \rangle }^{r}\,f(\omega )\\  &  & +{\langle \langle {\hat{d}}_{\sigma }(t);{\hat{d}}_{\sigma }^{\dagger }(\tau )\rangle \rangle }^{ < })\}\end{array}$$

Introducing the Nambu notation ${\hat{{\rm{\Psi }}}}_{d}^{\dagger }=({\hat{d}}_{\uparrow }^{\dagger },{\hat{d}}_{\downarrow })$, ${\hat{{\rm{\Psi }}}}_{d}={({\hat{{\rm{\Psi }}}}_{d}^{\dagger })}^{\dagger }$ we can define the matrix Green’s function $\,{G}_{d}(\tau ,\tau ^{\prime} )=\langle \langle {\hat{{\rm{\Psi }}}}_{d}(\tau );{\hat{{\rm{\Psi }}}}_{d}^{\dagger }(\tau ^{\prime} )\rangle \rangle$ and recast expression (12) as

13 $${I}_{i\uparrow }(V)=-\frac{2e{{\rm{\Gamma }}}_{N}}{h}\,\int \,d\omega \,{\rm{Im}}\,\{{\int }_{-\infty }^{t}\,d\tau {e}^{i(\omega -eV)(t-\tau )}({G}_{d}{(t,\tau )}_{11}^{r}\,f(\omega )+\,{G}_{d}{(t,\tau )}_{11}^{ < })\}$$

The lesser matrix Green’s function obeys the Keldysh equation $\,{G}^{ < }=(1+{G}^{r}\,{{\rm{\Sigma }}}^{r})\,{g}^{ < }(1+{G}^{a}\,{{\rm{\Sigma }}}^{a})+{G}^{r}\,{{\rm{\Sigma }}}^{ < }\,{G}^{a}$, where for brevity we dropped the temporal arguments. In our case the first term vanishes, so we are left with ${G}_{11}^{ < }={G}_{11}^{r}\,{{\rm{\Sigma }}}_{11}^{ < }\,{G}_{11}^{a}+{G}_{11}^{r}\,{{\rm{\Sigma }}}_{12}^{ < }\,{G}_{21}^{a}+{G}_{12}^{r}\,{{\rm{\Sigma }}}_{21}^{ < }\,{G}_{11}^{a}+{G}_{12}^{r}\,{{\rm{\Sigma }}}_{22}^{ < }\,{G}_{21}^{a}$. Using the explicit selfenergies ${{\rm{\Sigma }}}_{\alpha \beta }^{ < }(t,\tau )$ we finally obtain the total current given by^25^

14 $${I}_{i}(V)={I}_{i}^{0}(V)+{I}_{i}^{1}(V),$$

where the first contribution (Andreev current)

15 $${I}_{i}^{0}(V)=\frac{e}{h}\,\int \,d\omega \,{T}_{i}^{0}(\omega )\,[\,f(\omega +eV)-f(\omega -eV)]$$

describes processes, in which electrons from the normal STM tip are scattered back to the same electrode holes, injecting Cooper pairs to the superconducting substrate. Its transmittance depends on the anomalous (off-diagonal) retarded Green’s function

16 $${T}_{i}^{0}(\omega )={{\rm{\Gamma }}}_{N}^{2}\,{|{\langle \langle {\hat{d}}_{i\uparrow }{\hat{d}}_{i\downarrow }\rangle \rangle }_{\omega }^{r}|}^{2}+^{\prime} \uparrow \leftrightarrow \downarrow ^{\prime} .$$

The other contribution appearing in equation (14) takes the usual form

17 $${I}_{i}^{1}(V)=\frac{e}{\hslash }\,\int \,d\omega \,{T}_{i}^{1}(\omega )\,[f(\omega +eV)-f(\omega )]$$

and its transmittance consists of three terms

18 $$\begin{array}{rcl}{T}_{i}^{1}(\omega ) & = & {{\rm{\Gamma }}}_{N}\,{{\rm{\Gamma }}}_{S}{\rho }_{S}(\omega )\,({|{\langle \langle {\hat{d}}_{i\uparrow }{\hat{d}}_{i\uparrow }^{\dagger }\rangle \rangle }_{\omega }^{r}|}^{2}+{|{\langle \langle {\hat{d}}_{i\uparrow }{\hat{d}}_{i\downarrow }\rangle \rangle }_{\omega }^{r}|}^{2}\\  &  & -\frac{2{{\rm{\Delta }}}_{sc}}{|\omega |}\,{\rm{Re}}\,[{\langle \langle {\hat{d}}_{i\uparrow }{\hat{d}}_{i\uparrow }^{\dagger }\rangle \rangle }_{\omega }^{r}\,{\langle \langle {\hat{d}}_{i\uparrow }{\hat{d}}_{i\downarrow }\rangle \rangle }_{\omega }^{r}])+^{\prime} \uparrow \leftrightarrow \downarrow ^{\prime} \end{array}$$

with ${\rho }_{S}(\omega )=\frac{|\omega |}{\sqrt{{\omega }^{2}-{{\rm{\Delta }}}_{sc}^{2}}}\,\theta \,(|\omega |-{{\rm{\Delta }}}_{sc})$. These terms correspond to the single particle tunneling, electron to hole conversion (“branch crossing” in the language of Blonder-Tinkham-Klapwijk approach) and electron to Copper pair scattering, respectively^25^. At zero temperature ${I}_{i}^{1}(V)$ vanishes in the sub-gap regime *e*|*V*| < Δ_*sc*_ for this reason the charge current can be transmitted solely via the Andreev channel.

Situation studied by us in the main text is a bit more complex, because of the inter-site *p*-wave pairing that activates the equal spin Andreev scattering processes. Their contribution to the subgap current can be expressed in the same way as (15) with straightforward generalization of the transmission (16).

## References

[1] Kitaev AY (2001). Unpaired majorana fermions in quantum wires. Phys. Usp..

[2] He JJ, Ng TK, Lee PA, Law KT (2014). Selective equal–spin Andreev reflections induced by Majorana fermions. Phys. Rev. Lett..

[3] Sun H-H (2016). Majorana zero modes detected with spin selective Andreev reflection in the vortex of a topological superconductor. Phys. Rev. Lett..

[4] Hu L-H, Li C, Xu D-H, Zhou Y, Zhang F-C (2016). Theory for spin selective Andreev reflection in vortex core of topological superconductor: Majorana zero modes on spherical surface and application to spin polarized scanning tunneling microscope probe. Phys. Rev. B.

[5] Liu X, Sau JD, Das Sarma S (2015). Universal spin–triplet superconducting correlations of Majorana fermions. Phys. Rev. B.

[6] Ebisu H, Lu B, Taguchi K, Golubov AA, Tanaka Y (2016). Josephson current in a normal-metal nanowire coupled to a superconductor/ferromagnet/superconductor junction. Phys. Rev. B.

[7] Yuan NFQ, Lu Y, He JJ, Law KT (2017). Generating giant spin currents using nodal topological superconductors. Phys. Rev. B.

[8] Beiranvand R, Hamzehpour H, Alidoust M (2016). Tunable anomalous Andreev reflection and triplet pairings in spin–orbit–coupled graphene. Phys. Rev. B.

[9] Sticlet D, Bena C, Simon P (2012). Spin and Majorana Polarization in Topological Superconducting Wires. Phys. Rev. Lett..

[10] Kotetes P, Mendler D, Heimes A, Schön G (2015). Majorana fermion fingerprints in spin-polarised scanning tunnelling microscopy. Physica E.

[11] Szumniak P, Chevallier D, Loss D, Klinovaja J (2017). Spin and charge signatures of topological superconductivity in Rashba nanowires. Phys. Rev. B.

[12] Devillard P, Chevallier D, Albert M (2017). Fingerprints of Majorana fermions in current-correlations measurements from a superconducting tunnel microscope. Phys. Rev. B.

[13] Li, J., Jeon, S., Xie, Y., Yazdani, A. & Bernevig, B. A. The Majorana spin in magnetic atomic chain systems. *arXiv*:1709.05967 (2017).

[14] Jeon, S. *et al*. Distinguishing a Majorana zero mode using spin resolved measurements. *Science* **358**, 772 (2017). 10.1126/science.aan3670 29025997

[15] Nadj-Perge S (2014). Observation of Majorana fermions in ferromagnetic atomic chains on a superconductor. Science.

[16] Pawlak R (2016). Probing atomic structure and Majorana wave-functions in mono-atomic Fe-chains on superconducting Pb-surface. Qunatum Info.

[17] Ruby M (2015). End states and subgap structure in proximity-coupled chains of magnetic adatoms. Phys. Rev. Lett..

[18] Chevallier D, Klinovaja J (2016). Tomography of Majorana fermions with STM tips. Phys. Rev. B.

[19] Liu C-X, Sau JD, Das Sarma S (2017). Role of dissipation in realistic Majorana nanowires. Phys. Rev. B.

[20] Stanescu TD, Tewari S (2013). Majorana fermions in semiconductor nanowires: fundamentals, modeling, and experiment. J. Phys.: Condens. Matter.

[21] Maśka MM, Gorczyca-Goraj A, Tworzydło J, Domański T (2017). Majorana quasiparticles of inhomogeneous Rashba chain. Phys. Rev. B.

[22] Wu C-T, Anderson BM, Hsiao W-H, Levin K (2017). Majorana zero modes in spintronics devices. Phys. Rev. B.

[23] Mourik V (2012). Signatures of Majorana fermions in hybrid superconductor-semiconductor nanowire devices. Science.

[24] Zhang, H. *et al*. Ballistic Majorana nanowire devices. *arXiv*:1603.04069 (2016).

[25] Sun Q-F, Wang J, Lin T-H (1999). Resonant Andreev reflection in a normal metal – quantum dot – superconductor system. Phys. Rev. B.

